# Dynamic Frame Update Policy for UHF RFID Sensor Tag Collisions

**DOI:** 10.3390/s20092696

**Published:** 2020-05-09

**Authors:** Laura Arjona, Hugo Landaluce, Asier Perallos, Enrique Onieva

**Affiliations:** 1 Paul G. Allen Center for Computer Science and Engineering, University of Washington, Seattle, WA 98195, USA; 2 Faculty of Engineering, University of Deusto, 48007 Bilbao, Spain; hlandaluce@deusto.es (H.L.); perallos@deusto.es (A.P.); enrique.onieva@deusto.es (E.O.)

**Keywords:** Radio Frequency Identification (RFID), RFID sensor tag, EPC-global standard, anti-collision, tag estimation, frame update policy

## Abstract

The current growing demand for low-cost edge devices to bridge the physical–digital divide has triggered the growing scope of Radio Frequency Identification (RFID) technology research. Besides object identification, researchers have also examined the possibility of using RFID tags for low-power wireless sensing, localisation and activity inference. This paper focuses on passive UHF RFID sensing. An RFID system consists of a reader and various numbers of tags, which can incorporate different kinds of sensors. These sensor tags require fast anti-collision protocols to minimise the number of collisions with the other tags sharing the reader’s interrogation zone. Therefore, RFID application developers must be mindful of anti-collision protocols. Dynamic Frame Slotted Aloha (DFSA) anti-collision protocols have been used extensively in the literature because EPCglobal Class 1 Generation 2 (EPC C1G2), which is the current communication protocol standard in RFID, employs this strategy. Protocols under this category are distinguished by their policy for updating the transmission frame size. This paper analyses the frame size update policy of DFSA strategies to survey and classify the main state-of-the-art of DFSA protocols according to their policy. Consequently, this paper proposes a novel policy to lower the time to read one sensor data packet compared to existing strategies. Next, the novel anti-collision protocol Fuzzy Frame Slotted Aloha (FFSA) is presented, which applies this novel DFSA policy. The results of our simulation confirm that FFSA significantly decreases the sensor tag read time for a wide range of tag populations when compared to earlier DFSA protocols thanks to the proposed frame size update policy.

## 1. Introduction

Traditionally, Radio Frequency Identification (RFID) technology applications focused on item identification, location, and authentication. In the past years, the growing interest in wireless sensors has also reached RFID, and it has been transformed to a technology for both identification and sensing applications. As a result, RFID has become a crucial element of the Internet of Things (IoT) platform. Industry alliances, such as the NFC forum (for HF RFID) and the RAIN RFID alliance (for UHF RFID), have been formed to motivate and promote these efforts. The use of RFID technology to sense our physical world has expanded tremendously in the last decade. This has enabled the sensing ability of RFID technology to gather information from real-world objects and seamlessly integrate this data within the IoT.

RFID applications using wireless sensors require a fast communication protocol to read the sensor’s data, especially with increasing tag populations. The main purpose of the protocol is for the reader to first obtain the tag’s unique identification code, which is referred to as EPC, and then to receive the data from the sensors. The coexistence of several sensor tags sharing the reader’s interrogation zone provides RFID technology with great flexibility, but it does so at the expense of suffering from the tag collision problem. A collision occurs when two or more tags respond simultaneously to a reader command, which results in a waste of energy and an increase in the tag identification time and sensor data read time. Therefore, RFID systems require an anti-collision protocol to deal with tag collisions and to minimise their negative impact.

EPCglobal Class 1 Generation 2 (EPC C1G2) (ISO/IEC 18000-63) [1] is the standard currently adopted by RAIN, a fundamental component of the RFID market. This standard uses a Dynamic Frame Slotted Aloha (DFSA) protocol to handle the message collisions among RFID tags. A DFSA protocol is characterized by the strategy that it employs to update the transmission frame size alongside the identification and sensor data reading process. Most RFID manufacturers currently follow the EPC C1G2 standard. Consequently, this work focuses on DFSA protocols. In particular, we propose a novel frame update policy and the Fuzzy Frame Slotted Aloha (FFSA) protocol, which lower the time required for the reader to receive sensor data packets. FFSA meets EPC C1G2 requirements. Consequently, the proposed protocol can easily be deployed with current RFID technology infrastructure using commercial readers.

A great deal of effort has been paid to the problem of RFID sensor data collection. However, many existing solutions make the assumption that all tag EPCs in the system are known to the reader in advance [2,3,4]. However, this assumption is not true for most scenarios, where unexpected tags frequently appear [5]. Additionally, these solutions are not fully compliant with the EPC C1G2 standard, thus they cannot be implemented with existing commercial readers and sensor tags. For a protocol to be compliant with the EPC C1G2, it should employ the standard inventory and access commands to read the sensor data. Otherwise, commercial sensor tags [6] will not be able to interpret the commands and send a response back to the reader.

Several protocols have been presented in the literature that employ a DFSA strategy to address the collisions among tags’ messages and which are compliant with the EPC C1G, including Slot Counter [1], FuzzyQ [7], Chen14 [8], Eom [9], ILCM-SbS [10], ILCM-FbF [11], Chen16 [12], and SUBEB-Q [13]. Each protocol employs a different frame update strategy to receive the tag EPC and the sensor data, and it will be presented and compared in the next section. However, none of them has been designed with the focus of minimising the sensor data read time. This metric is critical given the increasing number of passive UHF RFID sensors sharing a common reader interrogation area.

### Novel Contributions over Prior Work

The major novelty of this paper is that we decrease the average time to receive the EPC and read a sensor data packet from one tag when compared with existing recent DFSA strategies that are compliant with the current standard. Consequently, the FFSA protocol is presented, which meets EPC C1G2 requirements. The main contributions made in this work are as follows:

1. An analysis and classification of the state-of-the-art DFSA tag anti-collision protocols according to their frame update policy.
2. A novel fast frame update policy for DFSA protocols. This policy first applies fuzzy logic to select the value of the slot where the frame size is updated. It then calculates the frame size as a function of the estimated number of tags inside the reader interrogation zone and the duration of the different time slots of the RFID platform.
3. We introduce the anti-collision Fuzzy Frame Slotted Aloha (FFSA) protocol, which applies the previous policy to lower the average time to read a sensor data packet from one tag compared with existing recent strategies.

The rest of this paper is organised as follows. Section 2 analyses the frame update policy of DFSA protocols. Next, Section 3 presents the related work and classifies the main state-of-of-art DFSA protocols according to their frame update policy. A novel frame update policy and the FFSA protocol are presented in Section 4. Section 5 provides the results of the performance evaluation followed by some of the limitations that we have identified. Finally, Section 6 concludes this paper and presents some recommendations for future work.

## 2. Analysis of Frame Update Policy of Dfsa Protocols

In order to improve different metrics regarding the process of tag identification, several DFSA anti-collisions protocols have been studied in the literature. Each strategy employs a different approach to update the frame size, with the focus of improving different performance metrics. Establishing a clear classification of all DFSA protocols is not straightforward. The key feature that differentiates DFSA protocols is the strategy that they follow to update the frame size. This section establishes a novel approach to classify the main frame update policies employed by DFSA anti-collision protocols. This classification considers three different perspectives to update *L*, which respond to the following three questions: how is *L* calculated? When is *L* examined? And, when must a new frame be started? The classification of the main up-to-date policies is summarised in Table 1. The literals in this table will be defined in the next section.

### 2.1. Frame Size Calculation

The reader adjusts *L* in each reading cycle according to the responses from the competing tags in each frame. Two main strategies can be found in the literature to set a value for the frame size in DFSA protocols: the first calculates *L* as a function of the parameter *Q*, and the second sets *L* as a function of the estimated number of tags $\hat{n}$. The parameter *Q* is an integer value used in the EPC C1G2 to set *L* as *L* = $2^{Q}$.

1. Parameter *Q*, f(*Q*): the frame size can be adjusted by controlling the number and types of the slots in each frame with the parameter *Q*, so that *Q* increases when collisions are detected and decreases with increasing number of idle slots. Several approaches in the literature update *L* by adjusting *Q* [1,7,14,15,16,17].
2. Tag set size estimation: several works in the literature have addressed the tag estimation task to provide an optimal frame size according to the estimated number of tags. It is known that a DFSA protocol reaches its maximum slot efficiency, which is defined as the ratio between the number of tags and the total number of slots required to identify them, when the frame size is equal to the number of tags. Therefore, to maximise this metric, the reader should set the frame size equal to the estimated number of tags. However, this condition of setting *L* = $\hat{n}$ is only satisfied if the reader assumes that the three types of slots have equal duration. However, the standard EPC C1G2 determines that each time slot has a different duration. Consequently, some approaches set the frame size according to $\hat{n}$ but assume unequal processing duration for each type of slot (single, collision, idle) [12,18].
  Once the tag set size has been estimated, the next step is to calculate *L* according to $\hat{n}$. Two main strategies to set *L* as a function of $\hat{n}$ can be found in the literature, which is presented next.
  - Continuous function of $\hat{n}$, f($\hat{n}$): the first strategy is to set *L* as a continuous function of $\hat{n}$. The reader analyses the information extracted from the tags’ responses and then sets *L* as a function of these values. Several anti-collision protocols follow this strategy [9,10,11,12,19,20,21,22,23,24].
  - Look-up table (LUT) according to $\hat{n}$, LUT($\hat{n}$): the second strategy is to set *L* according to an LUT based on $\hat{n}$. The idea is to define different ranges of $\hat{n}$ and assign a different value of *L* for each $\hat{n}$ range. Several approaches in the literature follow this strategy, including  [8,13,25,26,27,28].

### 2.2. Frame Size Examination

This section answers the question related to *when* (and in which slot) *L* must be examined, considering that an examination refers to a new calculation of *L*. DFSA algorithms update *L* dynamically. Therefore, a strategy is defined to establish in which slot or slots within a frame the value of *L* must be examined. Three main strategies can be found in the literature, as follows:

1. Frame by Frame (FbF): the simplest strategy is to only calculate *L* at the last slot of each frame [9,11,20,21,22,23,24,26,27].
2. Pointer by Pointer (PbP): some protocols have defined some particular slots within the frame, referred as the pointer *p* in the present paper (*p* < *L*), where *L* is examined to check its appropriateness [7,8,12,13,28]. These pointers are usually set as a fraction of the current frame size.
3. Slot by Slot (SbS): the third strategy is based on examining *L* at every slot of the frame [1,10,14,15,16,17,19,25].

### 2.3. Frame Break Condition

This section presents the different policies followed by the reader to decide, after calculating *L*, whether a new frame must be started, or if the reader must proceed with the next slot. Six main strategies can be found in the literature.

1. Different *L*: several protocols start a new frame when the new value of *L* differs from the current one [1,7,12,14,15,17,19,25,28].
2. *L* fits *n* from an LUT, LUT($\hat{n}$): some algorithms define an LUT based on $\hat{n}$ and *L* [8,13] to check the appropriateness of *L*. First, the reader searches in the LUT for the corresponding value of *L* for the previously obtained $\hat{n}$. Then, if this new value differs from the current one, a new frame is started. Otherwise, the reader proceeds to the next slot of the current frame.
3. Higher expected number of successful slots, $c_{s}\left(n,L\right)$: the authors in [10] define a policy to break the current frame and start a new one if the expected number of successful slots in the rest of the current frame ${c_{s}}_{1}\left(n,L\right)$ is less than that expected in the new frame ${c_{s}}_{2}\left(n,L\right)$. In other words, a new frame is started if ${c_{s}}_{2}\left(n,L\right)$ > ${c_{s}}_{1}\left(n,L\right)$.
4. Lower Identification Time, (lower $t_{IT}$): the authors in [16] present a frame cancellation strategy to minimise the total expected time to identify a tag set.
5. Lower sensor read time, (lower $t_{R}$): this work presents a strategy where a new frame is started if the expected average time for reading one sensor packet $t_{R}\left(n,L\right)$ in the new frame is lower than the one in the current frame.
6. End of Frame, (EoF): a new frame is started when the current frame has finished. This strategy is intrinsic to a DFSA-based anti-collision protocol and it is applied in all the protocols analysed in the present paper.

## 3. Related Work: Classification of Dfsa Protocols

In this section, we will present and classify some of the most relevant related work in DFSA protocols, including Slot Counter [1], FuzzyQ [7], Chen14 [8], Eom [9], ILCM-FbF [11], ILCM-SbS [10], Chen16 [12], and SUBEB-Q [13]. The performance evaluation of these protocols will be analysed and evaluated with detail in the next section, and will also be compared with the proposed solution FFSA.

**Figure 1:**
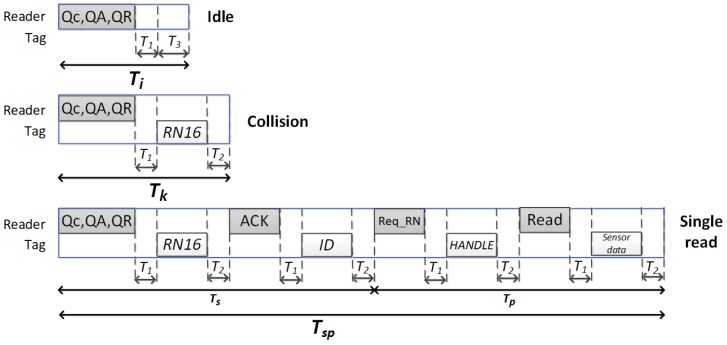
Transmission model of EPC Class 1 Generation 2 (C1G2).

The analysis performed in this work is based on the standard RFID wireless communication model, and it is shown in Figure 1. This figure shows the different reader and tags messages along with their corresponding duration meeting the EPC C1G2 requirements. A sequence of *L* slots is referred to as a *frame*, where *L* represents its size. The reader distinguishes between three different type of slots: idle (no tag respond), collision (two or more tags transmit a message simultaneously), and *single read* (the reader correctly receives the tag EPC during $T_{s}$ and one sensor data packet with during $T_{p}$). The duration of each slot is referred to as $T_{i}$, $T_{k}$, and $T_{sp}$, respectively. $T_{1}$, $T_{2}$, and $T_{3}$ separate the reader commands and tags responses.

Next, Table 2 presents a novel classification of the previous protocols, including the proposed FFSA. The classification is made according to the frame update policy followed by each protocol to identify a group of tags of size *n*.

## 4. The Proposed Frame Update Policy

This section introduces the novel fuzzy frame update policy. The arbitration of RFID communication is a stochastic process of unknown behaviour. Therefore, fuzzy logic is an efficient tool to model the process of identifying RFID tags. Fuzzy control for RFID anti-collision protocols was first introduced in [7], where a fuzzy system was used to give an intuitive value of the frame size. This work presents a Fuzzy Rule Based System (FRBS), which obtains the value of the pointer slot *p* to only accurately examine the frame size when appropriate. This solution is combined with a time-minimising function to update the value of *L* at slot *p*. The resulting proposed policy lowers the average time required to read one sensor packet from one tag compared to existing strategies. The three parts of the proposed policy (frame size calculation, frame size examination, and frame break condition) are presented next.

### 4.1. Frame Size Calculation to Minimise $T_{R}\left(N,L\right)$

The first part of the policy sets the value of *L* to minimise the expected time to receive one sensor data packet from one tag in a frame. For this purpose, the sensor data read time $t_{R}\left(n,L\right)$ is defined as the expected time to identify one tag among *n* in a frame of size *L* and read one sensor data packet:

(1) $$t_{R}\left(n,L\right)=\frac{\left(T_{s}+T_{p}\right)c_{s}\left(n,L\right)+T_{k}c_{k}\left(n,L\right)+T_{i}\;c_{i}\left(n,L\right)}{c_{s}\left(n,L\right)}$$

where $c_{s}\left(n,L\right)$, $c_{k}\left(n,L\right)$, and $c_{i}\left(n,L\right)$ are defined as the expected value of the number of single, collision, and idle slots in a frame, respectively. The duration of the slots, $T_{s}$, $T_{p}$, $T_{k}$, and $T_{i}$, are set according to the standard

(2) $$T_{s}=T_{command}+2T_{1}+2T_{2}+T_{RN16}+T_{ACK}+T_{EPC},$$

(3) $$T_{p}=T_{reqRN}+2T_{1}+2T_{2}+T_{handle}+T_{Read}+T_{data},$$

(4) $$T_{k}=T_{command}+T_{1}+T_{RN16}+T_{2},$$

and

(5) $$T_{i}=T_{command}+T_{1}+T_{3},$$

where $T_{command}$ is the duration of the reader command Qc, QA, or QR, referred as $T_{Qc}$, $T_{QA}$, and $T_{QR}$, respectively.

The parameters $T_{EPC}$ and $T_{RN16}$ correspond to the duration of the EPC and RN16 tag messages, respectively. These two parameters are calculated as a function of the Tag-to-Reader synchronisation time $T_{Preamble_{TR}}$, the length of each parameter, and the tag data rate $DR_{t}$, calculated as

(6) $$DR_{t}=1/BLF.$$

The parameter BLF refers to the Backscatter-link frequency. Thus,

(7) $$T_{RN16}=T_{Preamble_{TR}}+17bits/DR_{t},$$

(8) $$T_{EPC}=T_{Preamble_{TR}}+129bits/DR_{t},$$

(9) $$T_{handle}=T_{Preamble_{TR}}+38bits/DR_{t}$$

and

(10) $$T_{data}=T_{Preamble_{TR}}+118bits/DR_{t}.$$

The length of the sensor data packet $T_{data}$ is calculated by taking a commercial UHF RFID accelerometer sensor tag as a reference [6]. According to the sensor data sheet, each accelerometer data packet contains 10 bytes of data.

The reader transmits one QA or Qc command in the first slot of each frame. Then, it transmits consecutive QR commands in the following slots of the frame until it reaches the last slot of the frame. Assuming a frame with sufficiently large *L*, $T_{command}$ = $T_{QR}$ is applied in Equations (2), (4), and (5) when one frame is analysed.

The duration of the reader commands Qc, QA, QR, $Req_{RN}$, and ACK are calculated as

(11) $$T_{Qc}=T_{FSync_{RT}}+22bits/DR_{r},$$

(12) $$T_{QA}=T_{Preamble_{RT}}+9bits/DR_{r},$$

(13) $$T_{QR}=T_{Preamble_{RT}}+4bits/DR_{r},$$

(14) $$T_{reqRN}=T_{Preamble_{RT}}+40bits/DR_{r},$$

and

(15) $$T_{ACK}=T_{Preamble_{RT}}+18bits/DR_{r}.$$

The duration of the Read command $T_{Read}$ is calculated using a commercial UHF RFID accelerometer sensor tag as a reference [6]. Thus,

(16) $$T_{Read}=T_{Preamble_{RT}}+62bits/DR_{r}.$$

The parameters $T_{FSync_{RT}}$ or $T_{Preamble_{RT}}$ correspond to the Reader-to-Tag synchronisation time as defined in [1], and the reader data rate $DR_{r}$ is obtained as

(17) $$DR_{r}=1/\left(\left(T_{symbol_{0}}+T_{symbol_{1}}\right)/2\right),$$

where $T_{symbol_{0}}$ = Tari, and $T_{symbol_{1}}$ = 1.5·Tari. Tari represents the reference time interval for a symbol-0 (FM0 symbol) transmission.

Next, the value of *L* minimising $t_{R}\left(n,L\right)$ is obtained by evaluating an RFID system with *n* tags and one reader. In this system, we can apply a a binomial distribution $P_{r}\left(n,L\right)$ [9] to approximate the probability that *r* tags among *n* select one slot along a frame of size *L*

(18) Pr(n,L)=nr1Lr1−1Ln−r.

Additionally, $p_{s}\left(n,L\right)$, $p_{k}\left(n,L\right)$, and $p_{i}\left(n,L\right)$ correspond to the probabilities that only one tag, more than one tag or no tag, respectively, occupy a slot [7]. In order to obtain the expected number of idle, single, and collision slots in a frame with a size *L* sufficiently large, we can apply a Poisson distribution with mean ρ = *n*/*L* [9]. $c_{i}\left(n,L\right)$ is approximated with r=0 in Equation (18) by

(19) $$c_{i}\left(n,L\right)=Lp_{i}\left(n,L\right)=L{\left(1-\frac{1}{L}\right)}^{n}\approx Le^{-\rho }.$$

$c_{s}\left(n,L\right)$ is approximated with r=1 in Equation (18) by

(20) $$\begin{array}{cc} c_{s}\left(n,L\right) & =L\frac{n}{L}{\left(1-\frac{1}{L}\right)}^{n-1}\approx L\rho \left(\frac{n/\rho }{n/\rho -1}\right)e^{-\rho } \end{array}$$

Then, $c_{k}\left(n,L\right)$ is approximated by

(21) $$c_{k}\left(n,L\right)=Lp_{k}\left(n,L\right)=L\left(1-p_{0}-p_{1}\right).$$

By substituting Equations (19), (20), and (21) into (1), and applying $\frac{n/\rho }{n/\rho -1}$≈1, the following expression is obtained

(22) $$t_{R}\left(\rho \right)\approx \frac{\left(T_{s}+T_{p}\right)\rho e^{-\rho }+T_{i}e^{-\rho }+T_{k}\left(1-\left(1+\rho \right)e^{-\rho }\right)}{\rho e^{-\rho }}.$$

Computing the derivative of $t_{R}\left(\rho \right)$ in Equation (1) with respect to ρ yields

(23) $$\begin{array}{cc} \frac{dt_{R}\left(\rho \right)}{d\rho }\; & =\frac{T_{k}\left(e^{\rho }\left(\rho -1\right)+1\right)-T_{i}}{\rho^{2}}. \end{array}$$

Then, by posing $\frac{dt_{R}\left(\rho \right)}{d\rho }$ = 0, we obtain the following equation

(24) $$e^{\rho }\left(\rho -1\right)+1=\frac{T_{i}}{T_{k}}.$$

By solving Equation (24), the value of ρ that minimises $t_{R}\left(\rho \right)$ is obtained:

(25) $$\rho =1+W\left(\left(\frac{T_{i}}{T_{k}}-1\right)e^{-1}\right)$$

where W(x) is the Lambert W-function. Finally, the optimal frame size which minimises $t_{R}\left(n,L\right)$ is

(26) $$L=\frac{n}{\rho }$$

where ρ is obtained from Equation (25).

**Figure 2:**
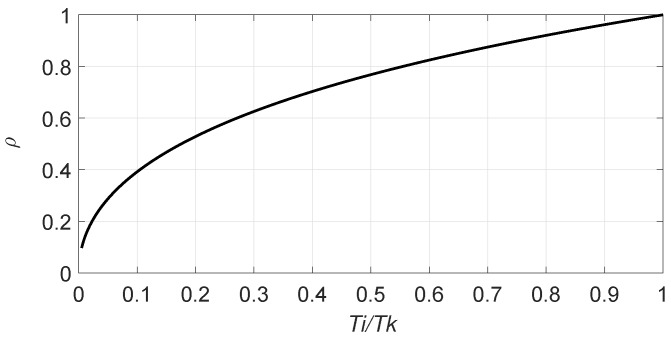
Evaluation of ρ solution in Equation (25) for $T_{i}/T_{k}$.

The value of ρ in Equation (25) is evaluated and presented in Figure 2 as a function of $T_{i}/T_{k}$. It can be appreciated that ρ decreases when the difference between $T_{i}$ and $T_{k}$ grows, which results in an increasing *L*. In conclusion, a higher difference in the values of $T_{i}$ and $T_{k}$ (with $T_{i}$ ≤ $T_{k}$) will result in a higher *L*. This result is coherent regarding the process of RFID tags identification and sensor data reading. If the duration of collision slots is much higher than that of idle slots, then it is necessary to increase *L* to reduce the number of collision slots. This occurs at the expense of an increase in the number of idle slots. However, because idle slots are much shorter than collision slots, this is an acceptable effect.

The previous analysis and Equation (26) demonstrate that the frame size calculation of the proposed policy is timing-aware. This means that the calculation is made as a function of the number of tags *n* and the timing parameters (ultimately the duration of the reader commands and tags responses) of the RFID scheme.

### 4.2. Frame Size Examination: Pbp

The second part of a frame update policy refers to the slot where *L* is examined. The FbF strategy is not efficient in the case of large frames filled with many collisions because the reader must wait until the frame has finished to update the frame size, which increases the identification time [11]. The SbS strategy involves the calculation of *L* at every single slot of the frame. As a consequence, one drawback of this solution is that it could overload a system with limited resources. Finally, the PbP strategy provides the flexibility of breaking the current frame before it ends, which maintains a low computational complexity in the reader. Therefore, the proposed policy applies a PbP strategy where the value of the pointer slot is dynamically updated using fuzzy logic.

**Figure 3:**
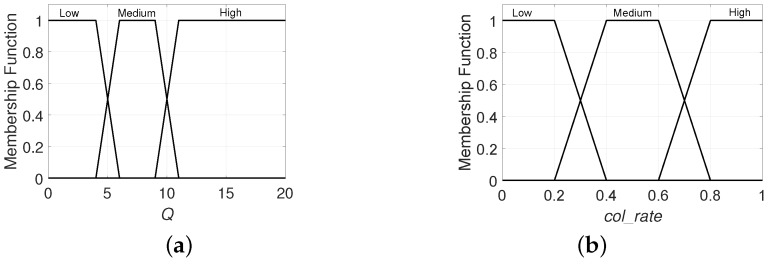
Membership functions: (**a**) for *Q*, (**b**) co_rate.

The proposed policy applies a fuzzy rule-based system (FRBS) to adjust the value of the pointer efficiently. Consequently, the current *L* and the tag collision rate co_rate are modelled as fuzzy sets to adaptively update the value of the pointer. A zeroth-order Takagi–Sugeno–Kang fuzzy system with a complete AND-composed rule [29] is proposed. The membership functions that we have used to codify the input variables are trapezoidal (see Figure 3) and the *t*-norm minimum is used to implement the AND operator. Among the traditional shapes of membership functions (triangular, trapezoidal, Gaussian, generalized bell, and sigmoid), trapezoidal membership functions have been selected due to their representation simplicity, which allows faster calculations. The proposed system has two inputs, as follows:

- *Q*: codifies the current value of this parameter which determines *L*, where Q∈N and 0≤Q≤20.
- co_rate: codifies the tag collision rate up to the current slot. This is defined by $co\_rate=c_{k}/slot\_index$, and 0≤co_rate≤1.

Additionally, the variable *slot_index* represents the reader’s internal counter, which keeps track of the present slot in the current frame.

The output *p* represents the slot where *L* must be examined. Specific values for membership functions and consequents in the rule base have been adjusted experimentally. The rules were designed also experimentally, considering the typical behaviour of an RFID system: on the one hand, more collisions (higher col_rate) require us to promptly examine *L* (smaller output *p*); while on the other hand, a smaller frame size (smaller *Q*) requires the examination of *L* in a later time slot (higher output *p*). The experimental values for the membership functions and the rules have been obtained by evaluating different ranges and selecting the one with the best performance in $t_{R}$.

**Figure 4:**
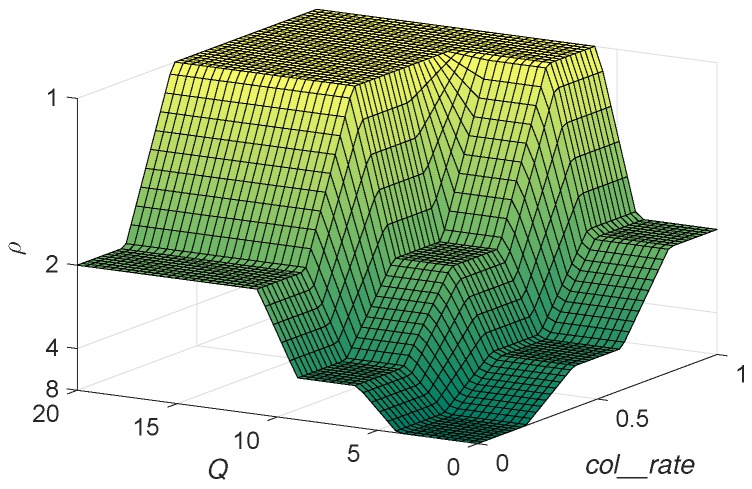
Surface representation of the proposed fuzzy rule-based system (FRBS), normalised to *L* = 16.

Figure 4 shows the surface representation of the proposed FRBS that determines the output *p*, normalised to *L* = 16. To illustrate an example, for the inputs *Q* = 10 and co_rate = 0.3, the output is *p* = *L*/9. Then, the new value of the pointer slot is *p* = round($2^{Q}$/9) = round($2^{10}$/9) = 114.

### 4.3. Frame Break Condition: Lower $T_{R}\left(N,L\right)$

Finally, the last part of the policy determines the condition to break the current frame and start a new one. The expected average time to read one sensor data packet [6] among *n* sensor tags in the current frame of size $L_{c}$ is obtained as   

(27) $$t_{R_{c}}=t_{R}{\left(n,L\right)|}_{n=\hat{n},L=L_{c}},$$

and the expected average time to read one sensor data packet among *n* sensor tags in the newly calculated frame of size $L_{n}$ is

(28) $$t_{R_{n}}=t_{R}{\left(n,L\right)|}_{n=\hat{n},L=L_{n}}.$$

To lower the tag sensor data read time, a new frame will be started if the condition $t_{R_{n}}$ < $t_{R_{c}}$ is satisfied. Thus, at slot *p*, the reader obtains $t_{R_{n}}$ and $t_{R_{c}}$ with Equations (27) and (28), assuming $T_{command}$ = $T_{QR}$, and then compares these values. Following this strategy, the reader guarantees that if a new frame is started at slot *p*, then the expected average time required to read one sensor data packet will be reduced.

### 4.4. The Proposed Fuzzy Frame Slotted Aloha Protocol

The novel FFSA protocol is introduced in this work, which applies the previously presented DFSA policy: determines the frame size minimizing $t_{R}\left(n,L\right)$ (Section 4.1), examines the frame size following a PbP strategy (Section 4.2), and starts a new frame with the condition to lower $t_{R}\left(n,L\right)$ (Section 4.3). FFSA is compliant with the EPC C1G2 standard, meaning that it meets the specific communication timing requirements and uses power-of-two values for *L*. As a consequence, this policy can be used to identify commercial sensor tags.

In order to calculate the frame size in Equation (26), FFSA applies the traditional Mean Minimum Square Error (MMSE) estimator [26] to calculate $\hat{n}$ as

(29) $$\hat{n}=\underset{n}{min}\left|\left(\begin{array}{c} p_{i}\left(n,L\right)L\\ p_{s}\left(n,L\right)L\\ p_{k}\left(n,L\right)L \end{array}\right)-\left(\begin{array}{c} c_{i}\\ c_{s}\\ c_{k} \end{array}\right)\right|.$$

MMSE has been applied in FFSA due to its computational simplicity while providing a relatively low estimation time.

The pseudocode of FFSA is presented in Algorithm 1. Initially, the reader sets the value of ρ with Equation (25) according to the RFID system timing parameters, and starts the identification procedure by broadcasting Qc. Each tag selects a slot in the frame to transmit its RN16, and the reader updates the variables $c_{s}$, $c_{k}$, and $c_{i}$ accordingly. When the reader reaches the last slot of the frame, the remaining tag population size is estimated with Equation (29). Then a new frame is started by broadcasting QA, specifying the new frame size as $Q_{n}=log_{2}\left(\left(\hat{n}-c_{s}\right)/\rho \right),L_{n}=2^{round(Q_{n})}.$ At every slot, col_rate is calculated and *p* is set as the current slot if col_rate = 1. If the current slot is a pointer, the reader calculates $\hat{n}$ with Equation (29) and sets $L_{n}$ with. Then, it obtains $t_{R_{c}}$ and $t_{R_{n}}$ with Equations (27) and (28). If the condition $t_{R_{n}}$ < $t_{R_{c}}$ is satisfied, a new frame is started and *p* is updated with the FRBS. Otherwise, the reader broadcasts QR to proceed to the next slot. The sensor tags reading process ends when there are no collision slots in the current frame and the frame is terminated.
**Algorithm 1:** Pseudocode of Fuzzy Frame Slotted Aloha (FFSA) protocol, reader operation. 1:Initialization: $L_{c}$=16, slot_index=1, calculate ρ with Equation (25)   2:Broadcast Qc 3:**while** 1 **do** 4: read slot and update $c_{i},c_{s},c_{k}$ 5: **if** slot_index = $L_{c}$ **and** $c_{k}=0$ **then** 6:  break 7: **end if** 8: **if** slot_index = $L_{c}$ **then** 9:  $\hat{n}=$MMSE($c_{s},c_{k},c_{i}$)  10:  $Q_{n}=log_{2}\left(\left(\hat{n}-c_{s}\right)/\rho \right)$, $L_{n}=2^{round(Q_{n})}$, $L_{c}$=$L_{n}$  11:  broadcast QA  12: **else**
13:  $col_{-}rate$ = $c_{k}$/slot_index  14:  **if** $col_{-}rate$=1 **then**15:   *p* = slot_index  16:  **end if** 17:  **if** slot_index = *p* **then**18:   $\hat{n}=$MMSE($c_{s},c_{k},c_{i}$)  19:   $Q_{n}=log_{2}\left(\left(\hat{n}-c_{s}\right)/\rho \right)$, $L_{n}=2^{round(Q_{n})}$  20:   **if** $t_{SR_{n}}$ < $t_{SR_{c}}$ **then**21:    *p* = FRBS($col_{-}rate$,$Q_{n}$), $L_{c}$=$L_{n}$  22:    broadcast QA23:   **else**
24:    slot_index = slot_index +1  25:    broadcast QR  26:   **end if**
27:  **else**
28:   slot_index = slot_index +1  29:   broadcast QR  30:  **end if** 31: **end if** 32:**end while**

## 5. Performance Evaluation

This section evaluates the performance of FFSA in terms of the average time to read one sensor data packet from one tag $t_{R}$. This metric is calculated as the total sensor read time divided by the total number of tags *n* in one inventory round:

(30) $$t_{R}=\frac{\left(T_{s}+T_{p}\right)c_{s_{T}}+T_{k}c_{k_{T}}+T_{i}c_{i_{T}}}{n}.$$

In one inventory round, the variables $c_{s_{T}}$, $c_{k_{T}}$, and $c_{i_{T}}$ are the total number of single, collision, and idle slot, respectively. The value of $T_{command}$ in Equations (2), (4), and (5) will vary depending on the slot position within a frame:

- First slot of the inventory round: $T_{command}$ = $T_{Qc}$.
- First slot of the frame: $T_{command}$ = $T_{QA}$.
- None of the above: $T_{command}$ = $T_{QR}$.

Table 3 summarizes the most relevant variables covered in this work. For each scenario, $t_{R}$ is evaluated as a function of the control variable indicated with *, *n* in S1 and BLF in S2. BLF is varied from 40 kbps (the minimum value allowed by the standard) to 640 kbps (maximum). S2 represents a special case because BLF also influences Tari, which represents the reference time interval for a data-0 transmission, and affects RTcal, TRcal, $T_{1}$, and $T_{2}$. These parameters are also modified every time that BLF changes during the simulation. In both scenarios, the initial *L* is set to 16. In FFSA, the initial value for *p* is set to eight and this value has been obtained experimentally. Table 4 shows the parameter values that we have employed.

Next, the protocols presented in Section 3 are evaluated and compared with FFSA for different performance metrics. A scenario with one reader and a varying number of tags has been evaluated with Matlab R2019, where the tags are uniformly distributed. The simulation responses have been averaged over 1000 iterations to ensure accuracy in the results. The performance evaluation followed in this work focuses on the media access control layer, ignoring the physical layer effects (assuming no capture effect and a non-impaired channel). This approach is widely accepted and incorporated by several studies in the related literature [8,10,11,12]. Our evaluation is performed for one inventory round, which is defined as the period of time that begins when the reader transmits the initial command Qc and which ends when the reader interrupts the reading process and the tags lose their state.

### 5.1. Impact of the Number of Tags in S1

**Figure 5:**
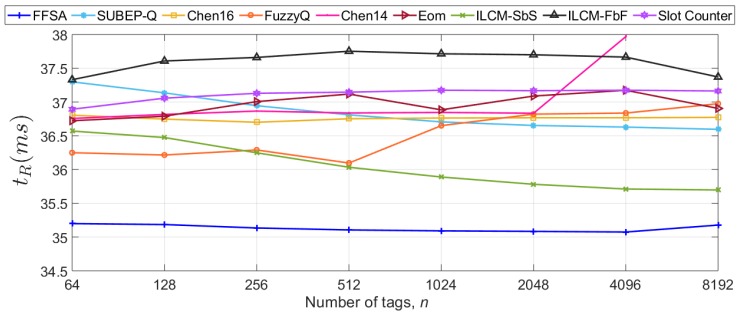
Evaluation results of the average time to read one sensor data from one tag in one frame in S1.

This section compares the selected protocols in terms of $t_{R}$ of Equation (30) by varying the number of tags *n* from 64 to 8192 (see Table 4). Additionally, $c_{k_{T}}$ and $c_{i_{T}}$ per tag are measured because $t_{R}$ is mostly influenced by them. The results of $t_{R}$ evaluation are shown in Figure 5. The average percentage improvement of FFSA compared to the rest of the protocols in terms of $t_{R}$ ranges from 3% to 9% in S1. This improvement will be more notable (above 9%) for shorter sensor data length (lower $T_{d}ata$).

**Figure 6:**
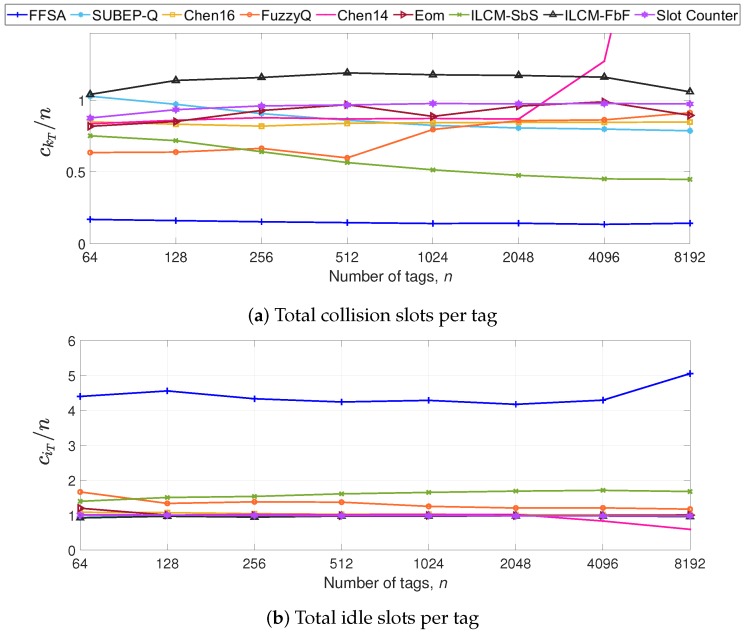
Evaluation results of $c_{k}$ per tag (**a**) and $c_{i}$ per tag (**b**) in S1.

Most protocols show a quasi-constant $t_{R}$ for *n* up to 2048 in Figure 5. FFSA requires the lowest average time read one sensor data packet from one tag. The strategy Chen14 shows an increasing $t_{R}$ for *n* > 2048 because it limits the frame size to 1024 when *n* is greater than 710. FuzzyQ also presents a peak at *n* = 2048, because the value of the *Q* parameter is upper-bounded. The improvement in FFSA comes from the reduction in $c_{k_{T}}$ at the expense of an increase in $c_{i_{T}}$, as can be appreciated in Figure 6a,b, respectively. Because the duration of an idle slot (Equation (5)) is shorter than that of a collision slot (Equation (4)), the reduction in $c_{k_{T}}$ leads to a lower $t_{R}$ for FFSA. The strategies ILCM-FbF and Chen14 present the highest $c_{k_{T}}$, leading to the highest $t_{R}$.

### 5.2. Impact of the Tag Backscatter Link Frequency in S2

This section compares the selected protocols in terms of $t_{R}$ (Equation (30)), while varying the tag Backscatter Link Frequency BLF. Therefore, the previous protocols are evaluated by varying BLF from 40 to 640 kbps; minimum and maximum values specified in the current standard. Tari is set to its minimum value 6.25 μs. The simulation results are averaged for *n* from 64 to 8192, and are shown iupn Table 5. The value of ρ employed by FFSA is also presented, which has been obtained with Equation (25).

All of the protocols present a decreasing $t_{R}$ with increasing BLF. For the highest values of BLF, all of the protocols present a similar behaviour and FFSA does not introduce a significant performance improvement. This occurs because the value of ρ (see Table 5) takes a significantly higher value, which causes a larger number of collision slots. As BLF decreases, FFSA shows a significant reduction in $t_{R}$ in relation to the prior protocols.

**Figure 7:**
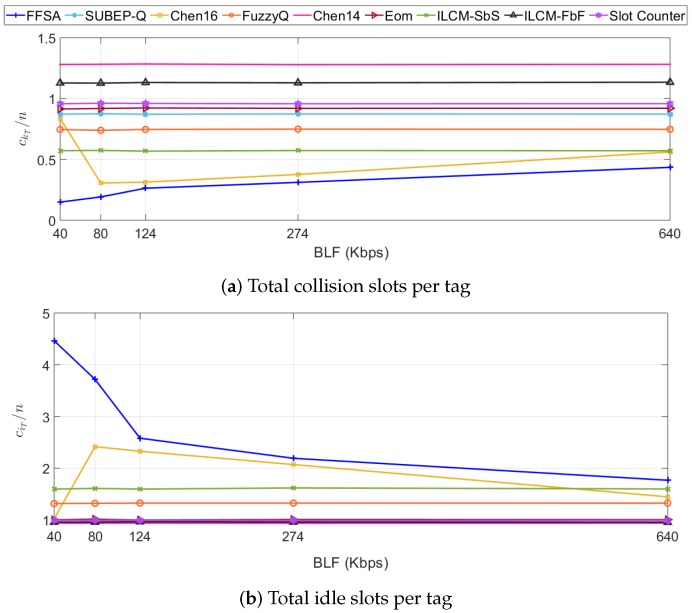
Evaluation results of $c_{k_{T}}$ per tag (**a**) and $c_{i_{T}}$ per tag (**b**) in S2.

To analyse the previous results, $c_{k_{T}}$ and $c_{i_{T}}$ per tag are measured as functions of BLF and averaged for all the tag set sizes *n* in S2, and the simulation results are shown in Figure 7. When BLF gets close to its upper limit, the increase in $c_{i_{T}}$ of FFSA is not compensated by the small reduction in $c_{k_{T}}$ in relation to the prior protocols, which limits the performance improvement of the proposed protocol. On the other hand, while the prior protocols present a quasi-constant $c_{k_{T}}$ with decreasing BLF, FFSA presents a notably decreasing $c_{k_{T}}$, which is reflected in a reduction in $t_{R}$ in relation to the prior protocols. Although for BLF > 80 kbps Chen16 behaves similarly to FFSA, the improvement introduced by FFSA becomes notably clear when BLF gets close to its upper or lower limit. This occurs because as BLF gets closer to its lower bound, Chen16 results in a low value of *y* (*y* is used by Chen16 in its algorithm to obtain *L*), which leads to an increasing $c_{k_{T}}$ and decreasing $c_{i_{T}}$.

### 5.3. Discussion

The previous section evaluated the performance of FFSA in terms of $t_{R}$ (Equation (30)). To demonstrate the benefits of the proposed protocol, its performance was compared with several related works presented in Section 3. In terms of the sensor data read time, the main parameter evaluated in this work, FFSA, presents the lowest $t_{R}$ for most of the values of *n* and BLF evaluated. The parameter BLF was selected as a control variable because it is related to the tag data rate. The two scenarios evaluated in this work consider that tags use Miller modulation with *M* = 4. The relationship between the tag BLF and the tag data rate is $DR_{t}$ = BLF/*M*. Thus, a higher BLF results in a faster tag, and vice versa. Consequently, the time to identify one tag $t_{R}$ is lower for higher BLF values. This effect is appreciated in Table 5. FFSA analyses this characteristic and takes into consideration the value of BLF to adjust *L* according to Equation (25). Therefore, FFSA lowers the sensor read time of the comparative protocols for a wide range of tag data rate configurations. In conclusion, the savings in the tag sensor data read time of FFSA is substantial for most of the range of BLF and *n*, which confirms that the proposed protocol is a time saving procedure in S1 and S2.

### 5.4. Identified Limitations

The protocols performance evaluation analysed in this work assumed an ideal communication channel, because it focused on the media access control layer. However, in a real scenario for passive RFID systems, the capture effect is typically present [30]. The capture effect occurs when the reader successfully resolves one tag reply in a collided slot. This effect could benefit the performance of FFSA because fewer collided slots and more single slots would occur, decreasing $t_{R}$. However, there is a negative impact of this effect over FFSA performance. The capture effect may hide some tags, which provides erroneous information to the tag estimator and increases the estimation error. Thus, the updated *L* value may not be appropriate, which negatively affects $t_{R}$. A study of the capture effect on $t_{R}$ and an evaluation of FFSA taking this effect into account is recommended for future work.

## 6. Conclusions

A comprehensive survey and classification of the frame update policies for RFID DFSA anti-collision protocols has been presented. In general, this policy can be divided into three parts: *L* update, *L* calculation, and frame break condition. Then, several state-of-the-art DFSA anti-collision protocols have been analysed and classified according to this policy. Finally, a novel frame update policy has been proposed. This results in the Fuzzy Frame Slotted Aloha (FFSA) protocol, which is a fast DFSA anti-collision protocol and is compliant with the current UHF RFID standard. With a significant improvement in the sensor data read time in relation to the current anti-collision protocols, FFSA is a suitable candidate where low sensor data read time is sought in UHF RFID systems that require a varying number of sensor tags.

## Figures and Tables

**Table 1:** Classification of Dynamic Frame Slotted Aloha (DFSA) frame update policies.

	Operation
*L* calculation	f(*Q*)
	f($\hat{n}$)
	LUT($\hat{n}$)
*L* examination	FbF
	SbS
	PbP
Frame break condition	Different *L*
	LUT($\hat{n}$)
	${c_{s}}_{2}$ > ${c_{s}}_{1}$
	Lower $t_{IT}$
	Lower $t_{R}$
	EoF

**Table 2:** Classification of main DFSA anti-collision protocols according to their frame update policy.

	L Calculation			L Exam		Frame Break Condition
	Type	$\hat{n}$, *Q*	*L*	Type	ρ	Type
Slot Counter [1]	f(*Q*)	*Q* = $Q_{fp}\pm C$	*L* = $2^{round(Q_{fp})}$	SbS	–	different *L* at slot or EoF
FuzzyQ [7]	f(*Q*)	*Q* = Q±ΔQ	*L* = $2^{round(Q)}$	PbP	*L*/9	different *L* at *p* or EoF
Chen14 [8]	LUT($\hat{n}$)	$\hat{n}=\left(c_{s}+2.39c_{k}\right)p$	LUT	PbP	*L*/4	LUT($\hat{n}$) at *p* or EoF
Eom [9]	f($\hat{n})$	$\hat{n}=\gamma c_{k}+c_{s}$	*L* = $2^{round(log_{2}\left(\hat{n}\right))}$	FbF	–	EoF
ILCM-FbF [11]	f($\hat{n}$)	$\hat{n}=kc_{s}+l$	*L* = $2^{round(log_{2}\left(\hat{n}\right))}$	FbF	–	EoF
ILCM-SbS [10]	f($\hat{n})$	$\hat{n}=kc_{s}+l$	*L* = $2^{round(log_{2}\left(\hat{n}\right))}$	SbS	–	Higher $c_{s}\left(n,L\right)$ at slot or EoF
Chen16 [12]	f($\hat{n})$	$\hat{n}=\left(c_{s}+2.39c_{k}\right)p$	*L* = $2^{round(log_{2}\left(y\hat{n}\right))}$	PbP	L/5	different *L* at *p* or EoF
SUBEB-Q [13]	f($\hat{n})$	$\hat{n}=\left(c_{s}+2.39c_{k}\right)p$	LUT	PbP	LUT	LUT($\hat{n}$) at *p* or EoF
FFSA	f($\hat{n})$	MMSE estimato [26]	*L* = $2^{round(log_{2}\left(\hat{n}/\rho \right))}$	PbP	FRBS	Lower $t_{R(n,L)}$ at *p* or EoF

**Table 3:** Main parameters used in the frame update analysis of this work.

Parameter	Description
*n*	Total number of tags
*L*	Transmission frame size
$\hat{n}$	Estimated number of tags
$T_{1}$, $T_{2}$, $T_{3}$	Link-timing parameters
$T_{i}$, $T_{sp}$, $T_{k}$	Duration of idle, single read, and collision slots
$T_{Qc}$, $T_{QA}$, $T_{QR}$, $T_{ACK}$$T_{req_{RN}}$, $T_{Read}$	Reader commands duration
$T_{RN16}$, $T_{EPC}$, $T_{handle}$, $T_{data}$	Tags messages duration
$c_{i}$, $c_{s}$, $c_{k}$	Number of idle, single, and collision slots in one frame
$c_{i_{T}}$, $c_{s_{T}}$, $c_{k_{T}}$	Number of idle, single, and collision slots in one inventory round
$P_{r}\left(n,L\right)$	Probability that *r* among *n* tags occupies a slot in a frame of size *L*
$p_{i}\left(n,L\right)$, $p_{s}\left(n,L\right)$, $p_{k}\left(n,L\right)$	Probability of idle, single, and collision slot in a frame of size *L*
$c_{i}\left(n,L\right)$, $c_{s}\left(n,L\right)$, $c_{k}\left(n,L\right)$	Expected value of the number of idle, single, and collision slots in one frame
$t_{R}$	Time to read one sensor data packet from one tag
$t_{R}\left(n,L\right)$	Expected time to read one sensor data packet from one tag among *n* in a frame of size *L*

**Table 4:** Parameters used in the simulations. * indicates the control variable.

Scenario	S1	S2
*n*	[64–8192] tags *	[64–8192] tags
BLF	40 kbps	[40–640] kbps *
Tari	6.25μs	6.25μs
RTcal	15.63μs	[15.63–62.50] μs
TRcal	17.34μs	[17.34–69.38] μs
$T_{1}$	24.50μs	[16.06–24.50] μs
$T_{2}$	375.50μs	[23.44–375.50] μs
$T_{3}$	15.63μs	15.63μs

**Table 5:** Effect of BLF and ρ on the protocols’ performance in terms of $t_{R}$ in S2. Quantities in bold represent the best results among the protocols in the comparison. * Indicates the control variable.

	$t_{R}$ (ms)				
BLF * (kbps)	**40**	**80**	**124**	**274**	**640**
ρ	**0.25**	**0.32**	**0.39**	**0.53**	**0.69**
FFSA	**35.14**	**18.30**	**12.30**	**6.30**	**3.46**
SUBEP-Q	36.84	19.04	12.73	6.46	3.50
Chen16	36.74	18.34	12.32	6.32	3.48
FuzzyQ	36.52	18.87	12.64	6.43	3.49
Chen14	38.01	19.63	13.13	6.65	3.59
Eom	36.97	19.10	12.78	6.48	3.51
ILCM-SbS	36.05	18.66	12.50	6.38	3.48
ILCM-FbF	37.57	19.40	12.98	6.58	3.56
Slot Counter	37.12	19.20	12.85	6.53	3.55

## References

[1] (2018). Radio Frequency Identity Protocols Class-1 Generation-2 UHF RFID Protocol for Communications at 860 MHz–960 MHz.

[2] Liu X., Cao J., Yang Y., Qu W., Zhao X., Li K., Yao D. (2019). Fast RFID sensory data collection: Trade-off between computation and communication costs. IEEE/ACM Trans. Netw..

[3] Qiao Y., Chen S., Li T., Chen S. (2016). Tag-ordering polling protocols in RFID systems. IEEE/ACM Trans. Netw..

[4] Chen S., Zhang M., Xiao B. Efficient information collection protocols for sensor-augmented RFID networks. Proceedings of the 2011 Proceedings IEEE INFOCOM.

[5] Muhammad S., Liu A.X. Expecting the unexpected: fast and reliable detection of missing RFID tags in the wild. Proceedings of the 2015 IEEE Conference on Computer Communications (INFOCOM).

[6] (2019). Farsens, EVAL01-Kineo-RM. http://www.farsens.com/wp-content/uploads/2018/07/DS-EVAL01-KINEO-RM-V05.pdf.

[7] Arjona L., Landaluce H., Perallos A., Onieva E. (2016). Fast fuzzy anti-collision protocol for the RFID standard EPC Gen-2. Electron. Lett..

[8] Chen W.-T. (2014). A fast anticollision algorithm for the EPCglobal UHF class-1 Generation-2 RFID standard. IEEE Commun. Lett..

[9] Eom J.-B., Lee T.-J. (2010). Accurate tag estimation for dynamic framed-slotted ALOHA in RFID systems. IEEE Commun. Lett..

[10] Solic P., Radic J., Rozic N. (2016). Early frame break policy for ALOHA-based RFID systems. IEEE Trans. Autom. Sci. Eng..

[11] Solic P., Radic J., Rozic N. (2014). Energy efficient tag estimation method for ALOHA-based RFID systems. IEEE Sens. J..

[12] Chen W.T. (2016). Optimal frame length analysis and an efficient anti-collision algorithm with early adjustment of frame length for RFID systems. IEEE Trans. Veh. Technol..

[13] Zhang G., Tao S., Cai Q., Gao W., Jia J., Wen J. (2019). A fast and universal RFID tag anti-collision algorithm for the Internet of Things. IEEE Access.

[14] Lee D., Kim K., Lee W. Q+-algorithm: An enhanced RFID tag collision arbitration algorithm. Proceedings of the 4th International Conference on Ubiquitous Intelligence and Computing.

[15] Daneshmand M., Wang C., Sohraby K. A new slot-count selection algorithm for RFID protocol. Proceedings of the Second International Conference on Communications and Networking in China.

[16] Zhu L., Yum T.-S. (2010). The optimal reading strategy for EPC Gen-2 RFID anti-collision systems. IEEE Trans. Commun..

[17] Teng J., Xuan X., Bai Y. A fast Q algorithm based on EPC Generation-2 RFID protocol. Proceedings of the 6th International Conference on Wireless Communications Networking and Mobile Computing.

[18] Porta T.F.L., Maselli G., Petrioli C. (2011). Anticollision protocols for single-reader RFID systems: Temporal analysis and optimization. IEEE Trans. Mob. Comput..

[19] Floerkemeier C., Wille M. Comparison of transmission schemes for framed ALOHA based RFID protocols. Proceedings of the International Symposium on Applications and the Internet Workshops.

[20] Schoute F. (1983). Dynamic frame length ALOHA. IEEE Trans. Commun..

[21] Cha J.-R., Kim J.-H. Dynamic framed slotted ALOHA algorithms using fast tag estimation method for RFID system. Proceedings of the 3rd IEEE Consumer Communications and Networking Conference.

[22] Chen W.-T., Lin G. (2006). An efficient anti-collision method for RFID System. IEICE Trans. Commun..

[23] Chen W.-T. An efficient scheme for multiple access in a RFID system. Proceedings of the 2006 International Conference on Wireless Networks.

[24] Chen W.-T. (2009). An accurate tag estimate method for improving the performance of an RFID anticollision algorithm based on dynamic frame length ALOHA. IEEE Trans. Autom. Sci. Eng..

[25] Joe I., Lee J. A novel anti-collision algorithm with optimal frame size for RFID system. Proceedings of the 5th ACIS International Conference on Software Engineering Research, Management Applications.

[26] Vogt H., Mattern F., Naghshineh M. (2002). Efficient object identification with passive RFID tags. Pervasive Computing.

[27] Wang B.S., Zhang Q.S., Yang D.K., Di J.S. Transmission control solutions using interval estimation method for EPC C1G2 RFID tag identification. Proceedings of the International Conference on Wireless Communications, Networking and Mobile Computing.

[28] Knerr B., Holzer M., Angerer C. Slot-by-slot minimum squared error estimator for tags populations in FSA protocols. Proceedings of the 2nd International EURASIP Workshop on RFID.

[29] Takagi T., Sugeno M. (1985). Fuzzy identification of systems and its applications to modeling and control. IEEE Trans. Syst. Man Cybern..

[30] Wu H., Zeng Y. (2015). Passive RFID tag anticollision algorithm for capture effect. IEEE Sens. J..

